# Contemporary prevalence estimates of undiagnosed and diagnosed atrial fibrillation in the United States

**DOI:** 10.1002/clc.23983

**Published:** 2023-03-01

**Authors:** Mintu P. Turakhia, Jennifer D. Guo, Allison Keshishian, Rachel Delinger, Xiaoxi Sun, Mauricio Ferri, Cristina Russ, Matthew Cato, Huseyin Yuce, Patrick Hlavacek

**Affiliations:** ^1^ Stanford University School of Medicine Stanford California USA; ^2^ Bristol Myers Squibb Lawrenceville New Jersey USA; ^3^ STATinMED, LLC Dallas Texas USA; ^4^ Pfizer New York City New York USA; ^5^ New York City College of Technology City University of New York New York City New York USA

**Keywords:** anticoagulant, atrial fibrillation, prevalence, stroke, systemic embolism

## Abstract

**Background:**

Atrial fibrillation (AF) prevalence estimates vary and have been based on cohorts with clinically established or diagnosed disease. Undiagnosed AF prevalence estimates are less certain as they are based on nongeneralizable convenience samples.

**Hypothesis:**

Because AF is often asymptomatic, it my remain undiagnosed until the development of complications such as stroke or heart failure. Consequently, the observed prevalence of diagnosed AF from the literature may underestimate total disease burden. We therefore sought to estimate the total prevalence of both diagnosed and undiagnosed AF.

**Methods:**

We performed a retrospective cohort study from 2012 to 2017 using data from five US medical claims data sets. Undiagnosed AF prevalence was estimated based on the observed incidence of ischemic stroke, systemic embolism (SE), and AF incidence after a stroke/SE. The diagnosed AF cohort included AF patients between Q1 2014 and Q3 2015. The undiagnosed AF cohort were patients with assumed undiagnosed AF in the year before a stroke/SE and who were newly diagnosed with AF in the 3‐month poststroke/SE. Stroke/SE incidence was calculated among all AF patients and the ratio of number of undiagnosed AF patients to stroke rate was created. Age‐ and sex‐adjusted estimates were stratified by period of assumed undiagnosed AF before poststroke/SE AF diagnosis (1 or 2 years).

**Results:**

The estimated US prevalence of AF (diagnosed and undiagnosed) in Q3 2015 was 5 628 000 cases, of which 591 000 cases (11%) were undiagnosed. The assumed 2‐year undiagnosed AF prevalence was 23% (1 531 000) of the total prevalent patients with AF (6 568 000). Undiagnosed (vs. diagnosed) AF patients were older and had higher CHA2DS2‐VASc scores. Of undiagnosed AF, 93% had CHA2DS2‐VASc ≥2 and met OAC criteria.

**Conclusions:**

These contemporary estimates demonstrate the high prevalence of undiagnosed AF in the United States. Undiagnosed AF patients are composed of primarily elderly individuals who if diagnosed, would meet criteria for stroke prevention therapy.

## Graphical abstract

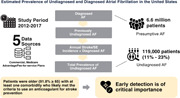

## 1 INTRODUCTION

Atrial fibrillation (AF) is the most common arrhythmia in the United States.^1^ AF is associated with considerable morbidity and mortality caused by related heart failure and ischemic stroke.^2^, ^3^

To date, estimates for AF prevalence have varied widely, ranging from approximately 1% to 2% of the US general population.^4^ However, these estimates have been primarily based on clinically diagnosed AF observed in cohort, survey, and claims analyses. Such estimates are impeded by challenges to AF diagnosis and likely to be underestimated, as many patients have paroxysmal, asymptomatic, or subclinical cases.^5^, ^6^ Screening has been mostly opportunistic to date; also, prior studies have been impeded by cost considerations and limited to small convenience samples that do not support generalizable overall prevalence estimates.^5^, ^7^

Another barrier to determining AF prevalence is the presence of undiagnosed AF.^8^ Undiagnosed AF cases are especially challenging as it is estimated that about a third of the AF population is asymptomatic.^9^ As such, it is often hospitalization for acute symptoms such as ischemic stroke that brings them to light.^5^ There is mounting evidence of considerable proportions of undiagnosed AF cases in the United States and Europe.^7^, ^8^, ^10^, ^11^ A previous study did provide a novel methodology for calculating the prevalence of AF, including those undiagnosed, in a large data set setting. This methodology back calculates undiagnosed AF prevalence by anchoring an AF diagnosis to a prior stroke, which is the main reason undiagnosed AF patients then get diagnosed.^8^, ^12^ However, these studies, including the one using the back‐calculation method, have been limited in size and study population generalizability. Thus, comprehensive characterization of the overall prevalence of undiagnosed AF, particularly in the United States, remains elusive.

To address this evidence gap, we undertook this study to estimate the prevalence of undiagnosed and diagnosed AF, through the application of the previously used back‐calculation methodology to population‐level data from a large and diverse integrated US commercial claims data set.

## 2 METHODS

### 2.1 Design and data sources

We performed a retrospective cohort study using integrated data from five medical claims data sets, with data from January 1, 2012, to December 31, 2017. The data sources included (1) the US Centers for Medicare & Medicaid Services (CMS) Medicare (fee‐for‐service); (2) IBM (formerly Truven) MarketScan® Commercial Claims and Encounter Database^13^; (3) IQVIA PharMetrics Plus™^14^; (4) Optum Clinformatics™ Data Mart^15^; and (5) Humana Research Database.^16^ The five integrated data sets were chosen for completeness, size, and complementary population heterogeneity—with precedent for our methodology.^17^, ^18^ Together, the data sets include claims from >163 million members of commercial and Medicare Advantage/fee‐for‐service plans, with demographics and enrollment history as well as medical claims from inpatient hospital, outpatient hospital, emergency room, physician's office, and surgery centers. Claims are coded using International Classification of Disease, 9th/10th Revisions, Clinical Modification (ICD‐9/10‐CM), Current Procedural Terminology, or Healthcare Common Procedure Coding System codes. To avoid duplication, we excluded patients with Medicare supplemental plans in MarketScan and PharMetrics data. Optum and Humana beneficiaries aged ≥65 years are enrolled in Medicare Advantage (Part C) plans and not covered in Medicare fee‐for‐service data, and all other beneficiaries are unique and therefore not duplicable across data sources. The IBM and IQVIA data sets contain information from employer‐provided health plans, with negligible potential duplication (0.5%).^19^

**Figure 1:**
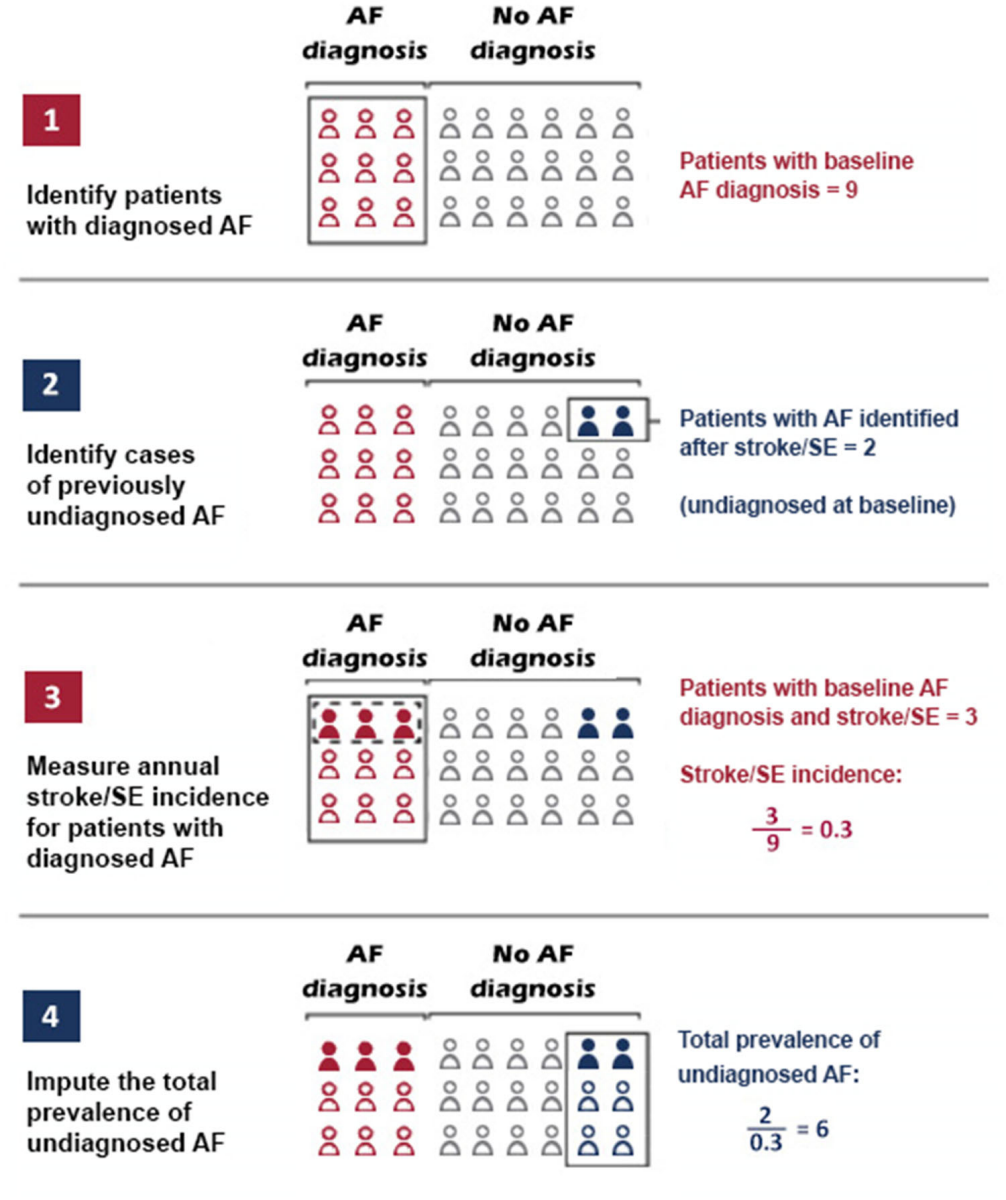
Steps for back‐calculation of the prevalence of undiagnosed atrial fibrillation. AF, atrial fibrillation; SE, systemic embolism.

**Figure 2:**
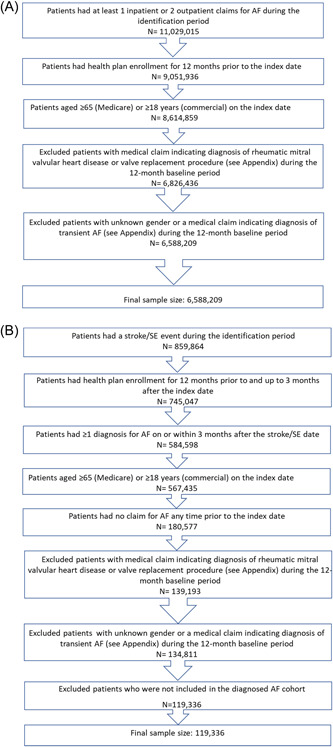
(A) Patient selection criteria for diagnosed atrial fibrillation patients. (B) Selection criteria for patients with AF diagnosed after a stroke/systemic embolism. AF, atrial fibrillation; SE, systemic embolism.

In each database, we calculated the prevalence of diagnosed and undiagnosed AF using a back‐calculation methodology that has been previously used.^8^, ^20^ In the first two steps, we created two cohorts to estimate the US prevalence of diagnosed and undiagnosed AF (the established AF and undiagnosed AF cohorts, respectively) (Figures 1 and 2A,B).

In the first step, we identified the established AF cohort which included patients with diagnosed AF who had ≥1 inpatient or ≥2 outpatient diagnoses (ICD‐9‐CM code 427.31; ICD‐10‐CM: I480‐I482, I4891, in any diagnosis position) between January 1, 2013, and December 31, 2017 (identification period; Step 1). The first AF diagnosis was designated as the index date. We excluded patients who were aged <65 years for the Medicare and <18 years for the four commercial databases as of the index date. Patients were required to have health plan enrollment for 12 months before and on the index date to evaluate baseline characteristics during the 12‐month baseline period (Figure 2A). Patients were also excluded if they had baseline evidence of transient AF (diagnosis of hyperthyroidism pericarditis, thyrotoxicity, or structural cardiac repair surgery) or valvular heart disease (heart valve replacement/mitral valve stenosis or a procedure for a valve replacement) (Supporting Information: Table 1).

In the second step, we identified the undiagnosed AF cohort which consisted of patients with AF identified only after a stroke/systemic embolism (SE) event during the identification period who were not in the established AF cohort. Stroke/SE events were defined as ≥1 hospitalization with a principal diagnosis of hemorrhagic or ischemic stroke or SE.^21^ The first stroke/SE hospitalization discharge date was designated as the index date. Patients were required to have health plan enrollment for 12 months before the index date (baseline period) through 3 months after the index date (AF identification period).^8^ To ensure stroke/SE association with newly diagnosed AF, patients were required to have an AF diagnosis in any position within the 3‐month postdischarge AF identification period and no previous AF claim any time before the index stroke/SE event (Figure 2B). Patients were also excluded if they had evidence of transient AF or valvular heart disease within the baseline period (Supporting Information: Table 1).

To estimate undiagnosed AF prevalence, we continued the back‐calculation method to evaluate the ratio of total patients diagnosed with AF after a stroke/SE event (denominator from Step 2) and stroke/SE risk among patients with AF (numerator) (Step 3 in Figure 1). We based this ratio on the assumption that because AF may cause stroke/SE (but the opposite is not the case), patients with stroke/SE and subsequent AF diagnosis had undiagnosed AF at the time of the stroke/SE event.

In Step 3, we measured the stroke incidence rates in each database among patients with diagnosed AF. To account for stroke/SE events not directly caused by AF, we determined AF incidence rates among patients with no prior AF diagnosis. We then divided the number of poststroke/SE AF patients in each quarter by the AF incidence during the identification period, to account for that all stroke/SE events may not be due to AF.

In Step 4, we estimated the total prevalence of undiagnosed AF as a function of the total patients diagnosed with AF after a stroke/SE event divided by the stroke/SE risk among patients with AF derived from Step 3.^8^ The raw results for each quarter were stratified by sex and age group and averaged to calculate an adjusted prevalence per quarter. In each database, the extrapolated undiagnosed prevalence was also stratified by age to represent the estimates for those populations both under and over 65 years of age. We then used US Census Bureau data to extrapolate the prevalence of diagnosed and undiagnosed AF to the US population, with adjustments for age and sex.^22^

To present a comprehensive range of estimates, we stratified all above‐mentioned analyses by periods of assumed prior undiagnosed AF, including: 1 year (4 quarters) before the poststroke/SE AF diagnosis; and 2 years (8 quarters) before the poststroke/SE AF diagnosis (Supporting Information: Figure 1). We conducted sensitivity analysis on patients with evidence of only ischemic stroke through Q3 of 2015 with the assumption of a 1‐year undiagnosed status.

In the established AF and undiagnosed AF cohorts, we conducted descriptive and comparative analysis of baseline variables—with means and percentages reported for continuous and categorical variables, respectively. Baseline variables included demographics (measured on the index date) and clinical characteristics (i.e., comorbidities and clinical risk scores (i.e., CCI, CHA_2_DS_2_‐VASc,^23^ modified HAS‐BLED) and healthcare utilization, which were measured during the 12‐month baseline period. *p* Values were calculated using *χ* ^2^ tests for categorical variables and *t* tests for continuous variables. Prevalence was reported as total counts and proportions per quarter. All analyses were performed using SAS® for Windows, Version 9.4 (SAS Institute).

## 3 RESULTS

### 3.1 Patient characteristics

Across all databases, we identified a total of 6.6 million patients with presumptive AF, 119 000 of whom with undiagnosed AF (Figure 2A,B). As compared with the newly diagnosed established AF cohort, patients in the undiagnosed AF cohort were more likely to be female, older, and significantly more likely to have a lower Deyo–Charlson Comorbidity Index score and a higher CHA_2_DS_2_‐VASc score. The undiagnosed AF cohort also had significantly higher incidence of baseline stroke yet lower healthcare resource utilization (Table 1). The results were generally consistent when stratified by age (<65 and ≥65 years) (Supporting Information: Table 2a,b).

**Table 1:** Baseline characteristics of patients with AF diagnosed before and after stroke.

	All patients with AF^a^	Newly diagnosed patients with AF^b^ (*n* = 3 997 403)		
		Established AF cohort	Undiagnosed AF cohort	
	Mean/%	Mean/%	Mean/%	*p* value
Sample size	6 588 209	3 878 067	119 336	
Age	77.5	76.8	78.6	<.0001
18–54	12.8%	14.8%	8.1%	<.0001
55–64	25.2%	26.0%	19.0%	<.0001
65–74	29.0%	30.7%	25.9%	<.0001
75–79	17.8%	17.4%	17.2%	.0556
≥80	45.0%	42.5%	49.7%	<.0001
Sex				
Male	51.7%	51.3%	46.2%	<.0001
Female	48.3%	48.7%	53.8%	<.0001
Geographic region				
Northeast	18.1%	17.6%	18.4%	<.0001
Midwest	25.6%	24.9%	24.3%	.0102
South	39.0%	39.6%	39.2%	.0033
West	17.4%	17.7%	16.8%	<.0001
Other	0.3%	0.4%	0.4%	.0702
Baseline comorbidity				
Deyo–Charlson Comorbidity Index	2.8	2.7	2.5	<.0001
CHA_2_DS_2_‐VASc score	4.0	3.8	4.1	<.0001
0	2.0%	2.5%	1.6%	<.0001
1	5.8%	7.0%	5.3%	<.0001
2	12.6%	14.6%	11.5%	<.0001
3	19.8%	21.2%	18.9%	<.0001
4+	59.7%	54.6%	62.7%	<.0001
HAS‐BLED score	2.8	2.7	2.8	<.0001
0	2.3%	2.8%	1.9%	<.0001
1	14.7%	17.8%	16.6%	<.0001
2	26.9%	26.2%	26.6%	.0044
3+	56.1%	53.2%	54.9%	<.0001
Baseline bleed	19.5%	17.6%	14.8%	<.0001
Stroke/SE	8.8%	7.5%	17.3%	<.0001
Congestive heart failure	22.8%	17.8%	13.3%	<.0001
Diabetes	33.9%	32.4%	33.9%	<.0001
Hypertension	77.1%	72.5%	75.5%	<.0001
Renal disease	20.0%	19.9%	19.1%	<.0001
Myocardial infarction	8.6%	8.3%	7.0%	<.0001
Dyspepsia or stomach discomfort	4.5%	6.3%	4.9%	<.0001
Peripheral arterial disease	23.7%	22.1%	21.9%	.0668
Transient ischemic attack	5.2%	5.2%	9.2%	<.0001
Coronary artery disease	37.0%	32.1%	27.6%	<.0001
Vascular disease	23.0%	22.6%	27.2%	<.0001
Baseline medication use				
ACE/ARB	14.6%	15.4%	14.8%	<.0001
Amiodarone	3.4%	1.8%	0.5%	<.0001
Beta‐blockers	32.3%	30.7%	30.6%	.3914
H2‐receptor antagonist	5.1%	5.4%	4.3%	<.0001
Proton pump inhibitor	21.4%	22.3%	17.7%	<.0001
Statins	36.8%	37.0%	33.4%	<.0001
Antiplatelets	11.6%	11.9%	11.8%	.6314
Electrocardiogram (ECG)	55.7%	50.4%	43.4%	<.0001
Holter monitor	5.0%	3.9%	2.0%	<.0001
External mobile cardiac telemetry monitor	0.9%	0.9%	0.3%	<.0001
Cardiac event monitor	1.3%	1.2%	0.4%	<.0001
Baseline all‐cause healthcare utilization				
Any outpatient visit	80.8%	77.5%	76.6%	<.0001
Any inpatient admission	25.8%	25.0%	19.4%	<.0001
Any pharmacy visit	69.8%	71.4%	67.2%	<.0001
No. of outpatient visits	19.15	16.47	14.43	<.0001
No. of inpatient admission	0.44	0.43	0.31	<.0001
No. of pharmacy visits	19.82	19.22	15.67	<.0001

Abbreviation: AF, atrial fibrillation.

^a^ All prevalent cases.

^b^ Incident cases only.

### 3.2 Prevalence

**Figure 3:**
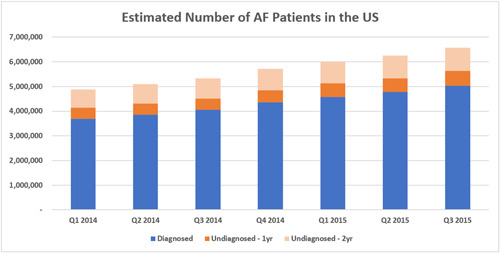
Estimated number of diagnosed and undiagnosed AF patients in the US 2014–2015. Prevalence is age‐ and sex‐adjusted to the US census. The estimate for assumed 2‐year undiagnosed status includes those who were included in the assumed 1‐year undiagnosed estimate. AF, atrial fibrillation.

After extrapolating the estimates for each quarter to the US population, we found that estimated quarterly period prevalence of AF increased from 3.7 million in Q1 2014 to 5.0 million in Q3 2015, with a progressive average increase of 5.0% per quarter (Figure 3).

**Figure 4:**
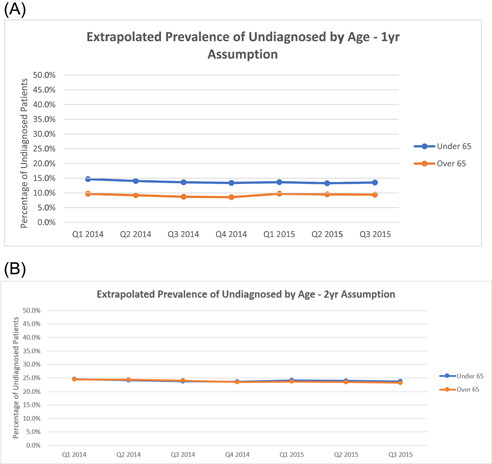
Estimated proportion of undiagnosed atrial fibrillation patients stratified by age and assumed undiagnosed period of: (A) 1 year (B) 2 years.

With the assumption of 1 year (4 quarters) of undiagnosed status in the undiagnosed AF cohort, we observed an increase in the number of undiagnosed cases per quarter—from 458 000 to 591 000 (Figure 3). This extrapolated to an overall undiagnosed AF prevalence estimate of 11% of 5.6 million total patients with AF (established plus undiagnosed cohorts) in Q3 2015. When stratified by age, the proportion of undiagnosed patients was slightly higher among those aged <65 years versus ≥65 years (Figure 4A).

With the assumption of 2 years (8 quarters) of undiagnosed status, we observed a similar increasing quarterly trend, ranging from 1.2 to 1.5 million (Q1 2014 and Q3 2015, respectively) (Figure 3). This extrapolated to an overall undiagnosed AF prevalence estimate of 23% of 6.6 million total patients with AF (both cohorts) in Q3 2015. When stratified by age, the proportions of undiagnosed patients were generally consistent (Figure 4B).

Sensitivity analyses conducted among patients with only ischemic stroke through Q3 2015 yielded results generally consistent with the main analyses. With the assumption of 1 year (4 quarters) of undiagnosed status, we estimated undiagnosed prevalence to be 11% of 5.6 million total patients with AF and evidence of only ischemic stroke in Q3 2015 (data not shown).

## 4 DISCUSSION

In this large, representative national sample, we estimated that the period prevalence of AF in Q3 2015 was 5 million patients. We estimated that another 600 000 to 1.6 million had undiagnosed AF. Based on these numbers, 11%–23% of an overall 5.6–6.6 million presumptive AF cases were considered undiagnosed, based on the assumptions of 1‐ (11%) and 2‐year (23%) undiagnosed status, respectively. Most of the undiagnosed patients were older and had a CHA_2_DS_2_‐VASc score of ≥2, making them eligible for oral anticoagulant treatment to mitigate stroke risk. These results suggest a considerable but addressable unmet need among this patient population.

Our findings are generally consistent with estimates in the literature and add more robust data to the evidence using multiple data sets over single‐source data sets previously used. Previous retrospective study estimates of total US AF prevalence have ranged from 5.2 to 5.3 million patients (2010 and 2009, respectively).^8^, ^24^ Another real‐world study estimated notably higher AF prevalence of 6.1 million in 2004 and 9.3 million in 2016.^25^ However, these discrepancies may be attributable to a much smaller sample of single‐center electronic medical record data with a predominantly white population. Our findings for undiagnosed patients with AF (11%–23%) also generally align with existing real‐world estimates ranging from 13.1% to 20%.^8^, ^10^, ^11^ The above‐mentioned 2009 US retrospective analysis estimated that of 5.3 million total patients with AF, ~700 000 (13.1%) were undiagnosed.^8^ Though the same methodology was used in this study as the prior study, this study uses more recent data, includes multiple data sources for increased generalizability, reports quarterly trends in undiagnosed AF and further describes those who were undiagnosed compared to the prior study. While there is a dearth of comparable US data, real‐world studies from France (2010) and Spain (2012) estimated undiagnosed proportions of 15% and 20%, respectively.^11^

Our study expands upon these analyses with additional, updated US evidence; a larger sample comprised of more databases; quarterly versus annual rates; and the use of stroke incidence in the modeling rather than CHADS_2_ scores. Moreover, our analysis affords observation of temporal trends in diagnosed and undiagnosed AF prevalence over nearly 2 years, and the consistent average quarterly increase of 5% speaks to the likelihood of increasing disease burden associated with undiagnosed AF. Results in the literature regarding temporal trends in AF prevalence are mixed, with some reports of increases but others of general stability.^24^, ^25^, ^26^, ^27^ Nonetheless, as the aging US population is a potential driver of increased prevalence, our study adds important data to the timeline.

Even with steady overall prevalence rates, the fact that undiagnosed AF prevalence is in line with our estimates could have important implications given the risk of AF‐related stroke and importance of early detection and treatment. The first of these is economic, as the incremental costs of undiagnosed AF in the United States has been estimated at $3.1 billion (2014 USD).^28^ Roughly estimated with the annual per capita incremental burden of $3616 derived from the above‐mentioned prior study, our results suggest the potential for associated incremental costs ranging from approximately $2.1 to $5.8 billion (2014 USD), much of which may be avoidable. A 2014 Medicare cost‐effectiveness analysis of Medicare patients with AF untreated with thromboprophylaxis found that a mere 10% increase in oral anticoagulant use among eligible but untreated patients would reduce AF‐related annual Medicare costs by 7.1%, or $187 million (per million total patients with AF; 2014 USD).^29^ For our undiagnosed population that would roughly be a range of approximately $112–$299 million within Medicare alone.

Our findings also highlight the potential for AF screening initiatives. Current clinical guidelines recommend active, opportunistic AF screening, and there is emerging evidence that systematic screening approaches can also improve detection.^30^, ^31^, ^32^ However, to date these approaches have been prone to small sample sizes and selection bias, and correcting these problems has been economically infeasible.^5^, ^7^, ^30^, ^33^ New screening approaches, such as mobile device applications, could potentially address these issues, although accuracy, feasibility, and penetration in the population can vary substantially.^34^, ^35^, ^36^, ^37^ Moreover, the US Preventive Services Task Force has recently concluded there is still insufficient evidence to assess the benefits of screening versus its costs.^32^ In addition, continued research on the role of screening in clinical outcomes will be necessary to further guide policy. Our results may help generate hypotheses for research that leverage claims data for this purpose and can inform future research to guide public health policy and treatment initiatives that address risk factors for AF.

### 4.1 Limitations

Several limitations are present in this analysis. First, our back‐calculation method assumes that patients with AF diagnosed after a stroke had AF for 1 or 2 years before the stroke event. This is likely given that AF is a chronic condition, but it is possible that patients would have undetected AF for varying lengths of time before diagnosis, impacting the results of the back‐calculation and extrapolated prevalence. Second, for the 2‐year estimate, we only required baseline enrollment for at least 1 year, so patients may have been diagnosed with AF before their health plan enrollment. However, 71% of patients had 2 years of medical enrollment before their AF diagnosis. Third, our back‐calculation method is novel and only previously implemented once, as such further validation of the method is needed to determine the accuracy of estimating undiagnosed AF.^8^ Fourth, it was assumed that the stroke rate in each database (1%–4%) was consistent for all patients and was accurately estimated. Fifth, the estimates rely on diagnosis codes and the accuracy of the back‐calculation method; therefore, screening studies would provide a better estimate of diagnosed and undiagnosed AF. Sixth, the back‐calculation relied on a diagnosis of AF after their stroke and may underestimate undiagnosed patients as patients could die before being diagnosed with AF, may not be symptomatic after their stroke and remain undiagnosed, or could have dominating neurologic issues that would make recoding an AF diagnosis a passing thought. Lastly, the prevalence calculation was based on insured patients with commercial, Medicare Advantage, or Medicare fee‐for‐service plans—this may not be generalizable to the entire US population, including those with other insurance plans (e.g., VA, Medicaid) and those without insurance. Patients without insurance are less likely to receive preventative care and services for chronic conditions and are more likely to die after an ischemic stroke compared to insured patients; therefore, our study may underestimate the prevalence and burden of undiagnosed AF.^38^, ^39^

### 4.2 Conclusions

Our study adds updated data to characterize undiagnosed AF at the national level in the United States. The data we generated from back‐calculation suggest considerable proportions of undiagnosed patients, in the context of growing overall prevalence, with diagnosed cases increasing from 3.7 to 5 million within 2 years and as many as 1.5 million presumptive undiagnosed cases at the end of that period. Together with the known burden of AF, this expanding unmet need underscores the critical importance of early detection. Our data can support both disease surveillance and future research and policy initiatives aimed at addressing this diagnostic gap.

## CONFLICT OF INTEREST STATEMENT

This study was sponsored by Pfizer and Bristol Myers Squibb. Mintu Turakhia reported receiving consulting fees from Pfizer, and outside of the submitted work, research grants from the ARISTA Alliance and Bristol Myers Squibb. Jennifer D. Guo was a paid employee of Bristol Myers Squibb at the time the study was conducted. Mauricio Ferri is a paid employee of Bristol Myers Squibb. Allison Keshishian, Rachel Delinger, and Xiaoxi Sun were paid employees of STATinMED, LLC at the time the study was conducted, which is a paid consultant to Pfizer and Bristol Myers Squibb in connection with the development of this manuscript. Cristina Russ, Matthew Cato, and Patrick Hlavacek are paid employees of Pfizer. Outside of the submitted work, Mintu Turakhia reports grants from Sanofi, American Heart Association, Food and Drug Administration, Gilead Sciences, Bayer, Bristol Myers Squibb, personal fees from Medtronic, Johnson & Johnson, AliveCor, Connect America, and Evidently. Mintu Turakhia is an employee of iRhythm Technologies, Inc. All work was completed before his employment at the company and was and continues to be done outside of this role.

## Supporting information

Supporting information.

## Data Availability

Due to restrictions in the data use agreements, the data will not be made available to other researchers.
