# Supplementary material for: Contemporary prevalence estimates of undiagnosed and diagnosed atrial fibrillation in the United States
Source: Clin Cardiol. 2023 Mar 1;46(5):484–93. doi: 10.1002/clc.23983 (PMC10189075; doi:10.1002/clc.23983)

**Supplemental Table 1. Selection Criteria Codes**

| **Inclusion Criteria Diagnoses** | | | |
| --- | --- | --- | --- |
| **Diagnosed AF** | | | |
| **Diagnosis** | | **ICD-9-CM Codes** | **ICD-10-CM Codes** |
| Atrial Fibrillation (AF) | | 427.31 | I480-I482, I4891 |
| **Undiagnosed AF** | | | |
| Stroke/SE | Hemorrhagic Stroke | 430.xx-432.xx. Cases will be excluded if traumatic brain injury (ICD-9: 800-804, 850-854) was present during hospitalization. | I6000, I6001, I6002, I6010, I6011, I6012, I602, I6030, I6031, I6032, I604, I6050, I6051, I6052, I606, I607, I608, I609, I610, I611, I612, I613, I614, I615, I616, I618, I619 |
|  | Ischemic Stroke | 433.x1, 434.x1, 436 | I6300, I63011, I63012, I63013, I63019, I6302, I63031, I63032, I63033, I63039, I6309, I6310, I63111, I63112, I63113, I63119, I6312, I63131, I63132, I63133, I63139, I6319, I6320, I63211, I63212, I63213, I63219, I6322, I63231, I63232, I63233, I63239, I6329, I6330, I63311, I63312, I63313, I63319, I63321, I63322, I63323, I63329, I63331, I63332, I63333, I63339, I63341, I63342, I63343, I63349, I6339, I6340, I63411, I63412, I63413, I63419, I63421, I63422, I63423, I63429, I63431, I63432, I63433, I63439, I63441, I63442, I63443, I63449, I6349, I6350, I63511, I63512, I63513, I63519, I63521, I63522, I63523, I63529, I63531, I63532, I63533, I63539, I63541, I63542, I63543, I63549, I6359, I636, I638, I639, I6789 |
|  | Systemic Embolism | 444.x, 445.x | I7401, I7409, I7410, I7411, I7419, I742, I743, I744, I745, I748, I749, I75011, I75012, I75013, I75019, I75021, I75022, I75023, I75029, I7581, I7589 |
| **Exclusion Criteria Diagnoses** | | | |
| **Diagnosis** | | **ICD-9-CM Codes** | **ICD-10-CM Codes** |
| Valvular Heart Disease | | 394.0, 394.1, 394.2, 394.9, 396.0, 396.1, 396.8, 396.9, 424.0, 745.xx | I050, I051, I052, I058, I059, I080, I088, I089, I340, I341, I342, I348, I349, Q213, Z952, Z953, Z954 |
| Valve Replacement Procedure | | 35.05-35.09, 35.20-35.28, 35.97 | 02RF07Z, 02RF08Z, 02RF0JZ, 02RF0KZ, 02RF37H, 02RF37Z, 02RF38H, 02RF38Z, 02RF3JH, 02RF3JZ, 02RF3KH, 02RF3KZ, 02RF47Z, 02RF48Z, 02RF4JZ, 02RF4KZ, 02RG07Z, 02RG08Z, 02RG0JZ, 02RG0KZ, 02RG37H, 02RG37Z, 02RG38H, 02RG38Z, 02RG3JH, 02RG3JZ, 02RG3KH, 02RG3KZ, 02RG47Z, 02RG48Z, 02RG4JZ, 02RG4KZ, 02RH07Z, 02RH08Z, 02RH0JZ, 02RH0KZ, 02RH37H, 02RH37Z, 02RH38H, 02RH38Z, 02RH3JH, 02RH3JZ, 02RH3KH, 02RH3KZ, 02RH47Z, 02RH48Z, 02RH4JZ, 02RH4KZ, 02RJ07Z, 02RJ08Z, 02RJ0JZ, 02RJ0KZ, 02RJ47Z, 02RJ48Z, 02RJ4JZ, 02RJ4KZ, 02UG3JZ, X2RF032, X2RF332, X2RF432 |
| Transient AF | | Hyperthyroidism: 242.x Coronary artery bypass surgery: 36.10, 36.19 Pericardial surgery: 37.10-37.12, 37.24, 37.25, 37.31-33, 37.35, 37.40 Structural cardiac repair surgery: 35.31-35.39, 35.41-35.42,35.50-35.54, 35.60-35.63, 35.70-36.73 | E0500, E0501, E0510, E0511, E0520, E0521, E0530, E0531, E0540, E0541, E0580, E0581, E0590, E0591, 0210083, 0210093, 02100A3, 02100J3, 02100K3, 02100Z3, 0210483, 0210493, 02104A3, 02104J3, 02104K3, 02104Z3, 02N60ZZ, 02N63ZZ, 02N64ZZ , 02N70ZZ, 02N73ZZ, 02N74ZZ, 02NK0ZZ, 02NK3ZZ, 02NK4ZZ, 02NL0ZZ, 02NL3ZZ, 02NL4ZZ, 02C60ZZ , 02C63ZZ, 02C64ZZ, 02C70ZZ, 02C73ZZ, 02C74ZZ, 02C80ZZ, 02C83ZZ, 02C84ZZ, 02C90ZZ, 02C93ZZ, 02C94ZZ , 02CK0ZZ, 02CK3ZZ, 02CK4ZZ, 02CL0ZZ, 02CL3ZZ, 02CL4ZZ, 02PA0YZ, 02PA4YZ, 02WA0YZ, 02WA3YZ, 02WA4YZ, 02CN0ZZ, 02CN3ZZ, 02CN4ZZ, 02NN0ZZ, 02NN3ZZ, 02NN4ZZ, 0W9D00Z, 0W9D0ZX, 0W9D0ZZ, 0WCD0ZZ, 0WCD3ZZ, 0WCD4ZZ, 02BN0ZX, 02BN3ZX, 02BN4ZX, 02BN0ZZ , 02BN3ZZ, 02BN4ZZ , 02TN0ZZ, 02TN3ZZ, 02TN4ZZ, 02B60ZZ, 02B63ZZ, 02B64ZZ, 02B70ZZ, 02B73ZZ, 02B74ZZ , 02BK0ZZ, 02BK3ZZ, 02BK4ZZ, 02BL0ZZ, 02BL3ZZ, 02BL4ZZ, 02560ZZ , 02570ZZ, 025K0ZZ, 025L0ZZ, 02B60ZZ, 02B70ZZ, 02BK0ZZ, 02BL0ZZ , 02T80ZZ, 028D0ZZ, 028D3ZZ, 028D4ZZ, 02QD0ZZ, 02QD3ZZ, 02QD4ZZ , 02890ZZ, 02893ZZ, 02894ZZ, 02Q90ZZ, 02Q93ZZ, 02Q94ZZ, 02QF0ZZ, 02QF3ZZ , 02QF4ZZ, 02QG0ZZ, 02QG3ZZ, 02QG4ZZ, 02QH0ZZ, 02QH3ZZ, 02QH4ZZ , 02QJ0ZZ, 02QJ3ZZ, 02QJ4ZZ, 02QA0ZZ, 02QA3ZZ, 02QA4ZZ, 02B50ZZ, 02B53ZZ, 02B54ZZ, 02RM0JZ, 02RM4JZ, 02U50JZ, 02U53JZ, 02U54JZ, 02UM0JZ, 02UM3JZ, 02UM4JZ, 024G0J2, 024J0J2, 02RM07Z, 02RM0KZ, 02RM47Z, 02RM4KZ, 02U507Z, 02U508Z, 02U50KZ, 02U537Z, 02U538Z, 02U53KZ, 02U547Z , 02U548Z, 02U54KZ, 02UM07Z, 02UM0KZ, 02UM37Z, 02UM3KZ, 02UM47Z, 02UM4KZ, 024G072, 024G082, 024G0K2, 024J072, 024J082, 024J0K2, 02RK07Z, 02RK0KZ, 02RK47Z, 02RK4KZ, 02RL07Z, 02RL0KZ, 02RL47Z, 02RL4KZ, 02U607Z, 02U608Z, 02U707Z, 02U708Z, 02U70KZ, 02U737Z, 02U738Z, 02U73KZ, 02U747Z, 02U748Z, 02U74KZ, 02UK0KZ, 02UK3KZ, 02UK4KZ, 02UL0KZ, 02UL3KZ, 02UL4KZ, 02Q50ZZ, 02Q53ZZ, 02Q54ZZ , 02QM0ZZ, 02QM3ZZ, 02QM4ZZ, 02QB0ZZ, 02QB3ZZ, 02QB4ZZ , 02QC0ZZ, 02QC3ZZ, 02QC4ZZ, 02BK0ZZ, 02NH0ZZ, 02RM0JZ, 02RP0JZ, 02RQ0JZ, 02RR0JZ, 02170ZP, 02170ZQ , 02170ZR, 02LS0ZZ, 02LT0ZZ, 02RM0JZ, 02U70JZ, 024F07J, 024F08J, 024F0JJ, 024F0KJ, 02S00ZZ, 02S10ZZ, 021L0ZW , 021L4ZW |

**Supplemental Table 2A. Baseline Characteristics of Patients <65 years**

|  | **Overall AF Patients** | **Newly Diagnosed (N=375,455)** | | |
| --- | --- | --- | --- | --- |
|  |  | **AF Diagnosed Before Stroke** | **AF Diagnosed After Stroke** |  |
|  | **Mean/%** | **Mean/%** | **N/Mean** | **P-value** |
| **Sample Size** | **538,025** | **366,759** | **8,696** |  |
| **Age** | 55.4 | 54.9 | 56.4 | <.0001 |
| **18-54** | 33.8% | 36.2% | 29.9% | <.0001 |
| **55-64** | 66.2% | 63.8% | 70.1% | <.0001 |
| **Gender** |  |  |  |  |
| **Male** | 68.7% | 67.0% | 64.0% | <.0001 |
| **Female** | 31.3% | 33.0% | 36.0% | <.0001 |
| **Geographic Region** |  |  |  |  |
| **Northeast** | 18.8% | 17.9% | 19.4% | 0.0005 |
| **Midwest** | 26.4% | 25.8% | 25.1% | 0.1440 |
| **South** | 40.1% | 41.7% | 42.6% | 0.0709 |
| **West** | 13.4% | 13.5% | 11.8% | <.0001 |
| **Other** | 1.3% | 1.1% | 1.1% | 0.4361 |
| **Baseline Comorbidity** |  |  |  |  |
| **Deyo-Charlson Comorbidity Index** | 1.5 | 1.5 | 2.0 | <.0001 |
| **CHA_2_DS_2_-VASc Score** | 1.5 | 1.4 | 1.9 | <.0001 |
| **0** | 24.9% | 26.4% | 21.5% | <.0001 |
| **1** | 33.3% | 33.1% | 25.3% | <.0001 |
| **2** | 22.8% | 22.1% | 21.1% | 0.0180 |
| **3** | 11.0% | 10.7% | 14.8% | <.0001 |
| **4+** | 7.9% | 7.7% | 17.4% | <.0001 |
| **HAS-BLED Score** | 1.3 | 1.3 | 1.6 | <.0001 |
| **0** | 28.1% | 29.5% | 25.9% | <.0001 |
| **1** | 33.8% | 31.8% | 27.4% | <.0001 |
| **2** | 22.2% | 21.8% | 22.7% | 0.0500 |
| **3+** | 15.9% | 16.8% | 24.0% | <.0001 |
| **Baseline bleed** | 13.1% | 13.1% | 14.7% | <.0001 |
| **Stroke/SE** | 3.9% | 3.5% | 18.0% | <.0001 |
| **Congestive Heart Failure** | 10.3% | 8.9% | 10.3% | <.0001 |
| **Diabetes** | 24.5% | 24.0% | 30.6% | <.0001 |
| **Hypertension** | 56.9% | 54.4% | 59.3% | <.0001 |
| **Renal Disease** | 8.0% | 8.5% | 11.8% | <.0001 |
| **Myocardial Infarction** | 5.4% | 5.7% | 8.2% | <.0001 |
| **Dyspepsia or stomach discomfort** | 4.6% | 6.1% | 5.8% | 0.2519 |
| **Peripheral Arterial Disease** | 7.5% | 7.5% | 12.5% | <.0001 |
| **Transient ischemic attack (TIA)** | 2.2% | 2.2% | 7.0% | <.0001 |
| **Coronary artery disease** | 19.6% | 18.1% | 20.6% | <.0001 |
| **Vascular disease** | 11.8% | 12.2% | 18.3% | <.0001 |
| **Baseline Medication Use** |  |  |  |  |
| **ACE/ARB** | 13.2% | 13.2% | 13.3% | 0.7996 |
| **Amiodarone** | 2.7% | 1.3% | 0.6% | <.0001 |
| **Beta blockers** | 30.9% | 26.2% | 24.7% | 0.0018 |
| **H2-receptor antagonist** | 3.2% | 3.4% | 3.5% | 0.5969 |
| **Proton pump inhibitor** | 18.8% | 18.8% | 17.9% | 0.0236 |
| **Statins** | 32.5% | 30.1% | 30.3% | 0.8164 |
| **Anti-platelets** | 7.0% | 7.3% | 10.6% | <.0001 |
| **Electrocardiogram (ECG)** | 52.9% | 48.2% | 44.1% | <.0001 |
| **Holter Monitor** | 5.9% | 4.7% | 1.9% | <.0001 |
| **External Mobile Cardiac Telemetry Monitor** | 0.7% | 0.6% | 0.1% | <.0001 |
| **Cardiac Event Monitor** | 3.4% | 3.0% | 0.6% | <.0001 |
| **Baseline All-cause Health Care Utilization** |  |  |  |  |
| **Any ER Visit** | 31.5% | 31.9% | 38.8% | <.0001 |
| **Any Office Visit** | 92.5% | 90.8% | 86.4% | <.0001 |
| **Any Outpatient Visit** | 95.8% | 94.7% | 91.1% | <.0001 |
| **Any Inpatient Admission** | 20.4% | 20.5% | 24.7% | <.0001 |
| **Any Pharmacy Visit** | 82.3% | 81.8% | 78.6% | <.0001 |
| **# of ER Visits** | 0.7 | 0.8 | 0.9 | <.0001 |
| **# of Office Visits** | 10.0 | 9.6 | 9.6 | 0.7784 |
| **# of Outpatient Visits** | 18.5 | 17.5 | 16.6 | 0.0009 |
| **# of Inpatient Admission** | 0.4 | 0.4 | 0.5 | <.0001 |
| **# of Pharmacy Visits** | 17.8 | 16.5 | 15.9 | 0.0020 |

**Supplemental Table 2B. Baseline characteristics of Patients ≥65 years**

|  | **Overall AF Patients** | **Newly Diagnosed (N=3,621,948)** | | |
| --- | --- | --- | --- | --- |
|  |  | **AF Diagnosed Before Stroke** | **AF Diagnosed After Stroke** |  |
|  | **Mean/%** | **Mean/%** | **Mean/%** | **P-value** |
| **Sample Size** | **6,050,184** | **3,511,308** | **110,640** |  |
| **Age** | 79.4 | 79.1 | 80.4 | <.0001 |
| **65-74** | 31.6% | 33.9% | 27.9% | <.0001 |
| **75-79** | 19.4% | 19.2% | 18.5% | <.0001 |
| **≥80** | 49.0% | 46.9% | 53.6% | <.0001 |
| **Gender** |  |  |  |  |
| **Male** | 50.2% | 49.6% | 44.8% | <.0001 |
| **Female** | 49.8% | 50.4% | 55.2% | <.0001 |
| **Geographic Region** |  |  |  |  |
| **Northeast** | 18.1% | 17.6% | 18.3% | <.0001 |
| **Midwest** | 25.2% | 24.2% | 23.9% | 0.2808 |
| **South** | 38.9% | 39.4% | 38.9% | 0.0013 |
| **West** | 17.7% | 18.2% | 17.2% | <.0001 |
| **Other** | 0.3% | 0.3% | 0.3% | 0.0001 |
| **Baseline Comorbidity** |  |  |  |  |
| **Deyo-Charlson Comorbidity Index** | 2.9 | 2.8 | 2.6 |  |
| **CHA_2_DS_2_-VASc Score** | 4.2 | 4.0 | 4.3 |  |
| **1** | 3.4% | 4.3% | 3.7% | <.0001 |
| **2** | 11.7% | 13.8% | 10.8% | <.0001 |
| **3** | 20.6% | 22.3% | 19.2% | <.0001 |
| **4+** | 64.3% | 59.5% | 66.3% | <.0001 |
| **HAS-BLED Score** | 2.9 | 2.9 | 2.9 |  |
| **0** | 0.0% | 0.0% | 0.0% |  |
| **1** | 13.0% | 16.3% | 15.8% | <.0001 |
| **2** | 27.4% | 26.7% | 26.9% | 0.1123 |
| **3+** | 59.7% | 57.0% | 57.4% | 0.0192 |
| **Baseline bleed** | 20.0% | 18.0% | 14.8% | <.0001 |
| **Stroke/SE** | 9.2% | 7.9% | 17.2% | <.0001 |
| **Congestive Heart Failure** | 23.9% | 18.7% | 13.6% | <.0001 |
| **Diabetes** | 34.7% | 33.3% | 34.1% | <.0001 |
| **Hypertension** | 78.9% | 74.4% | 76.8% | <.0001 |
| **Renal Disease** | 21.1% | 21.1% | 19.7% | <.0001 |
| **Myocardial Infarction** | 8.9% | 8.5% | 6.9% | <.0001 |
| **Dyspepsia or stomach discomfort** | 4.5% | 6.3% | 4.8% | <.0001 |
| **Peripheral Arterial Disease** | 25.1% | 23.7% | 22.7% | <.0001 |
| **Transient ischemic attack (TIA)** | 5.4% | 5.6% | 9.4% | <.0001 |
| **Coronary artery disease** | 38.6% | 33.6% | 28.1% | <.0001 |
| **Vascular disease** | 29.8% | 29.8% | 30.5% | 0.0183 |
| **Baseline Medication Use** |  |  |  |  |
| **ACE/ARB** | 14.7% | 15.6% | 15.0% | <.0001 |
| **Amiodarone** | 3.5% | 1.9% | 0.4% | <.0001 |
| **Beta blockers** | 32.4% | 31.2% | 31.1% | 0.3752 |
| **H2-receptor antagonist** | 5.2% | 5.6% | 4.3% | <.0001 |
| **Proton pump inhibitor** | 21.7% | 22.7% | 17.7% | <.0001 |
| **Statins** | 37.2% | 37.7% | 33.7% | <.0001 |
| **Anti-platelets** | 12.0% | 12.3% | 11.9% | <.0001 |
| **Electrocardiogram (ECG)** | 56.0% | 50.7% | 43.3% | <.0001 |
| **Holter Monitor** | 4.9% | 3.8% | 2.0% | <.0001 |
| **External Mobile Cardiac Telemetry Monitor** | 1.0% | 0.9% | 0.3% | <.0001 |
| **Cardiac Event Monitor** | 1.1% | 1.0% | 0.4% | <.0001 |
| **Baseline All-cause Health Care Utilization** |  |  |  |  |
| **Any ER Visit** | 31.6% | 30.9% | 31.3% | 0.0160 |
| **Any Office Visit** | 89.5% | 85.7% | 86.8% | <.0001 |
| **Any Outpatient Visit** | 93.5% | 90.2% | 92.0% | <.0001 |
| **Any Inpatient Admission** | 26.2% | 25.4% | 19.0% | <.0001 |
| **Any Pharmacy Visit** | 68.6% | 70.4% | 66.3% | <.0001 |
| **# of ER Visits** | 0.6 | 0.6 | 0.6 | <.0001 |
| **# of Office Visits** | 15.0 | 12.9 | 11.1 | <.0001 |
| **# of Outpatient Visits** | 19.2 | 16.4 | 14.3 | <.0001 |
| **# of Inpatient Admission** | 0.4 | 0.4 | 0.3 | <.0001 |
| **Length of Inpatient Stay (in days)** | 3.2 | 3.1 | 2.1 | <.0001 |
| **# of Pharmacy Visits** | 20.0 | 19.5 | 15.7 | <.0001 |

**Supplemental Figure 1. Study Design for Patients Diagnosed with AF after Stroke**
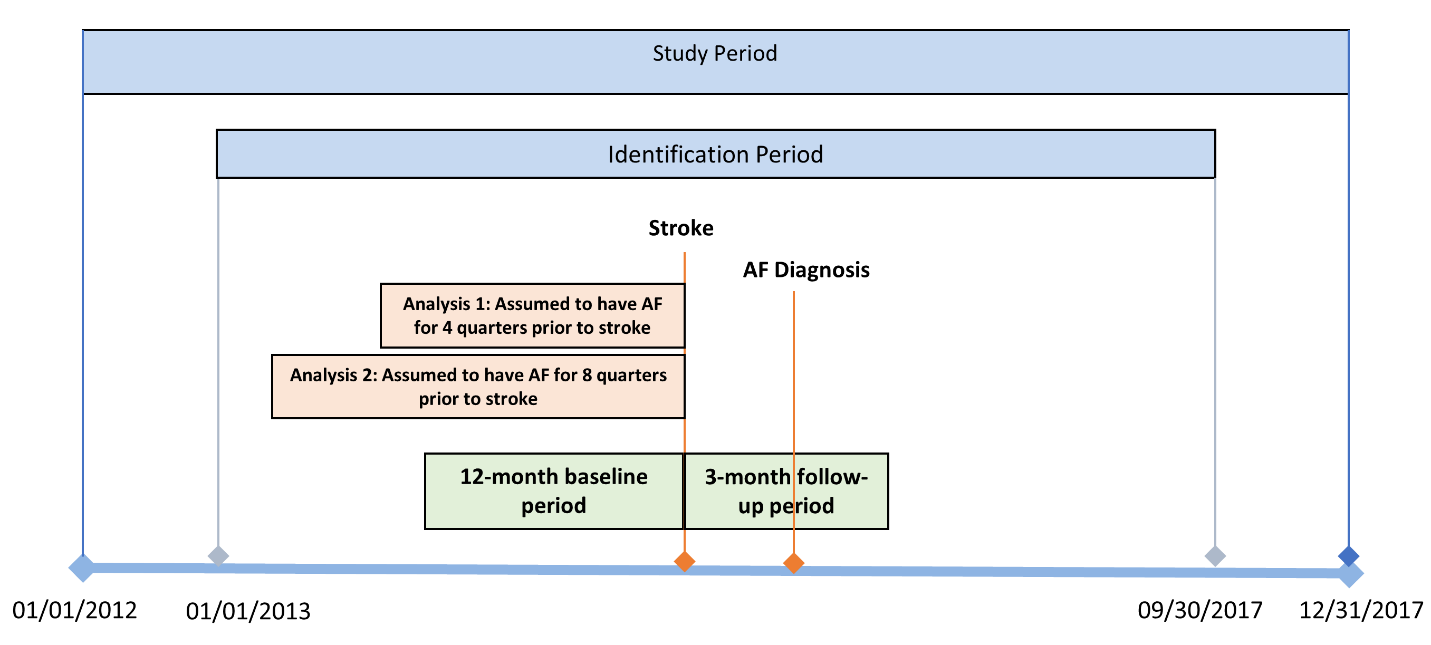

Supplement: Supplementary file 1 — Supporting information. [file CLC-46-484-s001.docx]
